# Supplementary material for: Foliar spraying exogenous ABA resists chilling stress on adzuki beans (Vigna angularis)
Source: PLoS One. 2024 Sep 9;19(9):e0304628. doi: 10.1371/journal.pone.0304628 (PMC11383210; doi:10.1371/journal.pone.0304628)
Supplement: S1 Data — (ZIP) [file pone.0304628.s001.zip › Data/Physiological indicators.docx]

**Table 3 Effect of LT stress and spraying ABA on SOD/CAT in leaf of adzuki bean**

| Varieties | Treatment code | | 1d | 2d | 3d | 4d | 5d |
| --- | --- | --- | --- | --- | --- | --- | --- |
| LXD 4  TJH | T1  T2  T3  T4  T5  CK  T1  T2  T3  T4  T5  CK | 1.42±0.10c  1.71±0.09bc  1.57±0.06c  1.41±0.06c  2.17±0.09a  1.97±0.12ab  1.88±0.16a  1.22±0.10b  1.67±0.07ab  1.73±0.17ab  1.92±0.23a  1.78±0.11a | | 1.13±0.06b  1.38±0.03b  1.34±0.05b  1.75±0.08a  1.86±0.04a  1.73±0.13a  1.29±0.07c  1.39±0.06bc  1.31±0.07c  1.51±0.02bc  1.88±0.12a  1.67±0.10ab | 0.99±0.01d  1.22±0.05cd  1.20±0.04cd  1.40±0.05c  1.90±0.05b  2.74±0.15a  1.13±0.01b  1.18±0.07b  1.10±0.05b  1.28±0.08b  2.26±0.26a  2.06±0.12a | 1.03±0.04d  1.24±0.02c  1.08±0.04d  1.33±0.02c  2.68±0.03a  1.94±0.06b  1.09±0.08b  1.17±0.07b  1.03±0.09b  1.20±0.13b  2.52±0.12a  2.90±0.26a | 1.13±0.02b  1.29±0.05b  1.29±0.07b  1.34±0.03b  2.17±0.08a  2.29±0.17a  1.23±0.10c  1.58±0.02bc  1.33±0.04bc  1.40±0.13bc  1.67±0.03b  2.52±0.22a |

**Table 4 Effect of LT stress and spraying ABA on SOD/POD in leaf of adzuki bean**

| Varieties | Treatment code | | 1d | 2d | 3d | 4d | 5d |
| --- | --- | --- | --- | --- | --- | --- | --- |
| LXD 4  TJH | T1  T2  T3  T4  T5  CK  T1  T2  T3  T4  T5  CK | 60.34±0.73c  69.72±0.20bc  54.61±0.64c  74.26±3.88ab  78.54±2.40a  77.29±0.68a  76.83±0.44c  83.46±1.14b  77.74±1.26d  83.37±0.89b  109.35±2.98a  107.26±1.20a | | 53.93±0.32d  61.47±1.53c  55.74±1.03d  69.29±0.57b  78.76±1.22a  79.72±1.84a  80.98±0.64b  78.95±1.21b  76.16±1.09b  81.93±1.86b  105.99±0.50a  102.04±3.86a | 54.39±0.39d  58.01±0.51c  55.70±0.35cd  68.34±0.83b  75.07±1.42a  73.31±0.78a  77.76±1.54b  80.88±0.73b  79.09±1.37b  80.02±1.62b  107.31±3.44a  100.75±4.15a | 54.24±0.54d  59.19±0.51c  55.76±0.37d  68.65±0.31b  77.83±0.75a  78.93±0.77a  79.07±1.07b  85.94±0.86b  80.67±1.02b  86.44±0.92b  107.91±4.49a  109.19±4.35a | 54.00±0.43c  57.33±0.17c  55.56±1.10c  66.21±0.51b  77.96±0.93a  76.39±1.67a  81.71±2.39b  86.03±1.21b  82.18±1.00b  88.52±1.19b  104.54±3.16a  100.05±2.62a |

**Table 4 Effect of LT stress and spraying ABA on SOD/POD in leaf of adzuki bean**

| Varieties | Treatment  code | **SOD/POD** | | | | |  | **SOD/CAT** | | | | |
| --- | --- | --- | --- | --- | --- | --- | --- | --- | --- | --- | --- | --- |
|  |  | 1d | 2d | 3d | 4d | 5d |  | 1d | 2d | 3d | 4d | 5d |
| LXD 4  TJH | T1  T2  T3  T4  T5  CK  T1  T2  T3  T4  T5  CK | 60.34  69.72  54.61  74.26  78.54  77.29  76.83  83.46  77.74  83.37  109.35  107.26 | 53.93  61.47  55.74  69.29  78.76  79.72  77.98  78.95  76.16  81.93  105.99  102.04 | 54.39  58.01  55.70  68.34  75.07  73.31  77.76  80.88  79.09  80.02  107.31  100.75 | 54.24  59.19  55.76  68.65  77.83  78.93  79.07  85.94  80.67  86.44  107.91  109.19 | 54.00  57.33  55.56  66.21  77.96  76.39  81.71  86.03  82.18  88.52  104.54  100.05 |  | 1.42  1.71  1.57  1.41  2.17  1.97  1.88  1.22  1.67  1.73  1.92  1.78 | 1.13  1.38  1.34  1.75  1.86  1.73  1.29  1.39  1.31  1.51  1.88  1.67 | 0.99  1.22  1.20  1.40  1.90  2.74  1.13  1.18  1.10  1.28  2.26  2.06 | 1.03  1.24  1.08  1.33  2.68  1.94  1.09  1.17  1.03  1.20  2.52  2.90 | 1.13  1.29  1.29  1.34  2.17  2.29  1.23  1.58  1.33  1.40  1.67  2.52 |

| Varieties | Treatment code | | 1d | 2d | 3d | 4d | 5d |
| --- | --- | --- | --- | --- | --- | --- | --- |
| LXD 4  TJH | T1  T2  T3  T4  T5  CK  T1  T2  T3  T4  T5  CK | 1.42  1.71  1.57  1.41  2.17a  1.97  1.88  1.22  1.67  1.73  1.92  1.78 | | 1.13  1.38  1.34  1.75  1.86  1.73  1.29  1.39  1.31  1.51  1.88  1.67 | 0.99  1.22  1.20  1.40  1.90  2.74  1.13  1.18  1.10  1.28  2.26  2.06 | 1.03  1.24  1.08  1.33  2.68  1.94  1.09  1.17  1.03  1.20  2.52  2.90 | 1.13  1.29  1.29  1.34b  2.17  2.29a  1.23  1.58  1.33  1.40c  1.67  2.52 |
