# Supplementary material for: Foliar spraying exogenous ABA resists chilling stress on adzuki beans (Vigna angularis)
Source: PLoS One. 2024 Sep 9;19(9):e0304628. doi: 10.1371/journal.pone.0304628 (PMC11383210; doi:10.1371/journal.pone.0304628)
Supplement: S1 Data — (ZIP) [file pone.0304628.s001.zip › Data/Yield.docx]

| Varieties | Treatment code | | 1d | 2d | 3d | 4d | 5d |
| --- | --- | --- | --- | --- | --- | --- | --- |
| LXD 4  TJH | T1  T2  T3  T4  T5  CK  T1  T2  T3  T4  T5  CK | 5.87±0.10b  5.24±0.09c  5.76±0.06bc  5.35±0.06bc  6.54±0.09a  5.77±0.12bc  4.13±0.02cd  3.89±0.07d  4.53±0.09bc  4.17±0.05bcd  5.85±0.22a  4.55±0.12b | | 5.27±0.07bc  4.54±0.12d  5.33±0.07bc  5.04±0.08cd  6.54±0.32a  5.77±0.15b  3.93±0.03cd  3.55±0.11d  4.30±0.06bc  3.55±0.12d  5.85±0.19a  4.55±0.12b | 4.77±0.01cd  4.15±0.05e  5.11±0.04c  4.50±0.05de  6.54±0.29a  5.77±0.19b  3.74±0.13cd  2.81±0.16e  3.98±0.04c  3.34±0.14d  5.85±0.25a  4.55±0.08b | 4.12±0.10cd  3.36±0.09e  4.36±0.04c  3.76±0.08de  6.54±0.22a  5.77±0.25b  3.20±0.20d  2.18±0.16e  3.84±0.10c  2.84±0.08d  5.85±0.26a  4.55±0.10b | 2.99±0.27c  2.23±0.04d  3.51±0.05c  2.83±0.10cd  6.54±0.35a  5.77±0.13b  2.51±0.13d  1.51±0.14e  3.22±0.14c  2.46±0.29d  5.85±0.30a  4.55±0.09b |
