# Supplementary material for: Foliar spraying exogenous ABA resists chilling stress on adzuki beans (Vigna angularis)
Source: PLoS One. 2024 Sep 9;19(9):e0304628. doi: 10.1371/journal.pone.0304628 (PMC11383210; doi:10.1371/journal.pone.0304628)
Supplement: Attachment — Submitted filename: Response to Reviewer.docx [file pone.0304628.s005.docx]

**Response to reviewer comments**

**Point 1.** Foliar spraying exogenous ABA resist chilling stress on Adzuki beans (*Vigna angularis*)

**RESPONSE: We have made changes to the title. (lines 1-2)**

**Point 2.** Write full name of AsA and GSH

**RESPONSE: As per suggestion, we added the full name of AsA and GSH (lines 39-40)**

**Point 3. “**Adzuki bean, one of the most important legume crops for human consumption, is cultivated in China, Korea, Japan, the Philippines, and other Southeast Asian countries” add references.

**RESPONSE: Thanks, we have added the references. (lines 47, 461-463)**

**Point 4. “**Low temperature adversely affects plant growth and metabolism, causing a large accumulation of reactive oxygen species (ROS) free radicals within plants” add references.

**RESPONSE: Thanks, we have added the references. (lines 56, 470-472)**

**Point 5. “**Among them, low temperature during flowering is the most harmful.” Why and add references.

**RESPONSE: Thanks, we have rewritten the sentences and added references. (lines 62-69, 473-485)**

**Point 6.** Shows statistical differences among the values of different treatments. (lime 218)

**RESPONSE: Thanks, we reworked the data and corrected errors. (lines 210-222)**

**Point 7.** Shows statistical differences among the values of different treatments (Table 3). (lime 308)

**RESPONSE: Thanks, we reworked the data and corrected errors, and modified the results analysis section. (lines 299-307, 308-Table 3)**

**Point 7.** “Its core mechanism involves diminishing reactive oxygen species accumulation, reducing membrane peroxidation, and minimizing cell membrane damage. The main stress response is to enhance the activity of antioxidant enzymes, increase the content of non-enzymatic antioxidant substances, etc., to adjust the reduction of MDA and H_2_O_2_ content and reduce the accumulation of O_2_^—·^and other ROS substances, and ultimately mitigate the impact of chilling damage on the yield of adzuki beans” add supporting references.

**RESPONSE: Thanks, we have added the references. (lines 339, 535-542)**

**Point 8.** “Spraying exogenous ABA can significantly increase the grain weight per plant of adzuki beans after low-temperature stress by increasing the enzyme activity of the AsA-GSH cycle and effectively alleviating the impact of low temperature on yield (Figure 9).” add supporting references.

**RESPONSE: Thanks, this is partly a summary based on our experiments. (lines 339)**
