# Supplementary material for: Foliar spraying exogenous ABA resists chilling stress on adzuki beans (Vigna angularis)
Source: PLoS One. 2024 Sep 9;19(9):e0304628. doi: 10.1371/journal.pone.0304628 (PMC11383210; doi:10.1371/journal.pone.0304628)
Supplement: Attachment — Submitted filename: Response to Reviewer-1.docx [file pone.0304628.s006.docx]

**Response to reviewer comments**

Thank you for your patience and for the insightful comments. I would like to express my sincere apologies for overlooking the comments in the PDF attachment during our initial revision process. It was an unintentional oversight, and I assure you it was not our intention to disregard the valuable feedback provided. Upon realizing this mistake, we promptly reviewed the comments and have addressed each point meticulously in our latest revisio.

**Point 1.** The results are described descriptively, maybe it is better to express some results quantitatively.

**RESPONSE:** **Thank you very much for your comments. We have add quantitative description in the abstract. (lines 40-41)**

**Point 2.** When the name of the compounds is mentioned in the text for the first time, write the full name and then use the abbreviation.

**RESPONSE: Thanks very much for your comments. As per suggestion, we added the full name of AsA and GSH (line 40)**

**Point 3.** lt is better that the keywords are not exactly the same as the title words.

**RESPONSE: Thanks, we have made changes to the title. (lines 1-2)**

**Point 4.** Please change this title to something more appropriate

**RESPONSE: Thanks, We are very sorry for the poorly written. We have changed the title as “Leaf spray treatment”. (line 87)**

**Point 5.** Please state with which software the correlation plot was drawn

**RESPONSE: Thank you very much for your comments.** **We are sorry for neglecting this point, in the** **revised manuscript, we have** **made the changes as suggestion. (lines 149-150)**

**Point 6**. lsn't it better to use 4 times or 3.8 times?

**RESPONSE: Thanks, in the revised manuscript,** **we have made the changes as suggestion. (lines 158)**

**Point 7.** The average comparison test and the statistical level of the study should be added below the figures and tables.

**RESPONSE: Thank you for your comments. We are sorry that we did not write clearly, we have made the changes as suggestion. (lines 169-170, 196-197, 214-215, 229-230, 232, 264-265, 271-272, 313-314)**
